# Treatment of Irregularity

**Published:** 1872-06

**Authors:** G. W. Munhall


### Treatment of Irregularity.

BY G. W. MUNHALL.

In this age of great things there is a danger of forgetting the more essential—the little things. It is a matter of great gratification to me when I hear of some one of the prominent men of the profession grappling with and obtaining the mastery over some great scientific problem; and, although such discoveries may not always be utilized so that we who are less versed in the lore of the schools can appropriate them to our practical use, yet, nevertheless, they often prove doorways through which we enter and roam, to us, hitherto untrodden fields. I am benefited as well as gratified, when I hear of the successes that have been made by my brethren in the profession in matters of common practice, and *how* such successes have been achieved. The REGISTER has given much space to such experiences, and, I think, 'tis well. Thinking that, possibly, I may be able to interest and benefit some humble plodder, I herewith submit the following case:

Mrs. R——, *ætat* 29 (twenty-nine), of nervo-lymphatic temperament. came to me about three years ago to ask my advice about a tooth that had caused her no little annoyance. The tooth—the right superior lateral incisor—stood so far inside the line of the other teeth—fully a half inch—that it could not be seen by a person standing immediately before her. It was, otherwise, an annoyance both in eating and speaking. The space it should occupy was just large enough for it. She said she had consulted a dentist in a distant city and also one in this city, and that they had both given it as their opinion that "it might have been done when she was in her teens, but she was too old now."

I told her that I could move the tooth into its proper place, and, it was my opinion that if it could be held there long enough, either the palatine wall of the alveolar process would follow it up, or an osseous formation would accumulate about the palatine surface of the tooth of sufficient strength to hold it permanently in position.

She expressed an anxiety to have me test my theory, and promised faithfully to obey to the letter all my directions. I took an impression of the mouth, and made a plate of vulcanite, allowing it to extend over the grinding surface of the molars, and pass around in front of the bicuspids, cuspids, and incisors, careful that it should fit the labial surface of the same closely, allowing the band to arch outward a little, opposite the tooth I desired to move. I may add, however, that before vulcanizing, I placed the wax plate in the mouth, and had the patient bite into the wax until the teeth came as nearly together as I desired they should, and, besides, obtained by this means an impression of the inferior molars.

I vulcanized in a Hay's vulcanizer at a temperature of 280°, for an hour and ten minutes. This gave me a tough and flexible plate. In finishing the same I rounded the outer edges of the band—which, of course, was more bulky than a gold one, but no more unsightly, and being closely fitted to the teeth was much firmer than a gold one could possibly have been made—so that it in no way interfered with the movements of the upper lip. I drilled two holes through this band opposite the tooth to be operated on, through which I passed silken floss, which I attached to a rubber band I had placed around the tooth. By drawing this thread tightly, and changing the rubber bands from time to time for one thicker, I was enabled to apply a very considerable amount of force.

I had the patient call every other day, each time increasing the force applied. At the end of four weeks the tooth began to move, and within two months from the time I first adjusted the plate I had it in its proper position. I then made another plate of the same material, allowing it to fit the lingual surface of the teeth closely, and leaving it, where it came in contact with the teeth, about half a line in thickness, except where it fitted against the tooth I had moved, at which point I let the plate cover the entire lingual surface. The next day after adjusting the plate the patient left the city to be gone about a month. I gave her the necessary instructions how to remove and replace the plate when she desired to cleanse it. After having been absent about a week, she carelessly left the plate out of her mouth one evening upon retiring to rest, and in the morning found the tooth back in its old quarters. She hastened back to the city in great alarm. Adjusting plate number one, I very easily moved the refractory incisor into line again, after which I placed plate number two in position as before. By directions the patient called again in six months. I found the tooth quite loose; and, as she said the plate gave her no trouble whatever—in fact she never thought of it except when it was necessary that it should be cleansed. I dismissed her with the request that she would call again at the end of six months. She did as requested, and, as I found the tooth quite firmly in its place, the indications of its former location entirely obliterated, which strengthened me in the belief that the palatine wall of the alveolus could be made to follow the tooth. I removed the plate and dismissed her with directions that if there was any evidence of a change in the position of the tooth to report in person forthwith. In about a month the patient returned, and, upon examination, I found that the tooth had gone back about a line. I brought it out to its place again, and, adjusting plate number two, dismissed her with the request that at some distant day she should call again. I saw nothing of her for a year. She had been wearing the plate all this time. She remarked that she would just as soon wear it as not, as it occasioned no inconvenience whatever. I found the tooth in its proper place, apparently as immovable as any of its fellows. I removed the plate, and have seen nothing of the lady from that time until the other day, when she called at my office. I found the tooth securely in position, just where I wanted it.

With reference to the intimation I made that the process might follow the tooth, I have this to say:

The process was unusually prominent. The palatine wall at this point was quite thin. In making the plates I cut away the casts, so that, when adjusted in the mouth, there was considerable pressure brought to bear on this part of the process. I continued this pressure by bending the plate at this point from time to time. In my judgment the operation was not successful, wholly because of this—only in part, for I believe that neither the tooth or palatine wall of the process would have been permanently secured in their proper positions, as they are now, if there had not been a pretty liberal deposit of osseous matter.

